# Acute tubulointerstitial nephritis associated with infliximab therapy in a patient with Crohn’s disease: a case report

**DOI:** 10.1007/s13730-024-00943-6

**Published:** 2024-11-02

**Authors:** Naif Alghamdi, Fahad Alshehri, Sultan Alhazza, Fahad Bhutto, Azhari Alhassan, Mohammed Kechrid, Dhafer Alshehri, Kadi Alshammari, Talal Assiri, Ohoud Assiri, Emad Darewsh, Mohammed Ali, Ruba Qadri, Yasser Alahmadi

**Affiliations:** 1 Internal Medicine Department, Nephrology Division, Security Forces Hospital Program, Riyadh, Saudi Arabia; 2 Internal Medicine Department, Security Forces Hospital Program, Riyadh, Saudi Arabia

**Keywords:** Acute tubulointerstitial nephritis, Crohn’s disease, Infliximab

## Abstract

We report the case of a 39-year-old man who presented with a history of generalized fatigue, nausea, subjective fever with rigors, and renal dysfunction after receiving infliximab (IFX) therapy for Crohn’s disease. A renal biopsy revealed acute tubulointerstitial nephritis (ATIN). After other causes of acute kidney injury were excluded, steroid therapy was initiated, his fever subsided, and kidney function improved. From this case report, infliximab could be a rare cause of elevated kidney function and that it should be not considered a completely safe treatment or disregarded as potential cause of ATIN.

## Introduction

Acute interstitial nephritis (AIN) is an acute kidney inflammation characterized by cellular and fluid exudation in interstitial tissue [1]. Renal manifestations vary in inflammatory bowel disease (IBD), including nephrolithiasis (calcium oxalate and uric acid), glomerulonephritis (immunoglobulin A [IgA] nephropathy and antineutrophilic cytoplasmic antibody-associated vasculitis [AAV]), renal amyloidosis (amyloid A), drug-related nephrotoxicity, and tubulointerstitial disease (acute tubular injury and tubulointerstitial nephritis) [2]. In contrast, in IBD determine whether AIN is an extraintestinal manifestation or whether it occurs secondary to medical treatments with drugs such as aminosalicylic acids (5-ASA) is difficult to determine. Tumor necrosis factor-alpha (TNF-α) inhibitors, such as infliximab (IFX) and adalimumab, are proinflammatory cytokines that are effective against moderate and severe Crohn’s disease and ulcerative colitis. Renal complications resulting from anti-TNF-α therapy are rare. Here, we report the case of a patient with Crohn’s disease who developed ATIN after the first dose of IFX and managed with steroid.

## Case presentation

A 39-year-old white Saudi male was diagnosed in August 2023 with stricturing/fistulizing (enteroenteric fistula) Crohn’s disease (Montreal score, A2L1B2) based on clinical imaging and colonoscopy findings complicated by small bowel obstruction. The patient required intensive care unit admission and started receiving total parenteral nutrition. Treated with a tapering dose of prednisolone and 50 mg of azathioprine orally once daily (PO OD), Laparoscopic ileocecal resection with end ileostomy were performed. However, the procedure was complicated by a large lower abdominal collection measuring 18 × 21 × 7 cm, which was drained, and Ciprofloxacin therapy was administered for 14 days. A follow-up abdominal CT scan with intravenous contrast showed regression of the abdominal collection. In intensive care unit, patient developed Aki secondary to sepsis from PICC line but declined after hydration and infection treatment, returned to normal within 1 month, and remained normal upon discharge. patient had no known medical or surgical histories. His family history was positive for IBD, but negative for kidney disease. The patient’s kidney function remained normal before and after the imaging, surgery, and antibiotic treatment.

On May 6, 2024, the patient received the first dose of biosimilar IFX (5 mg/kg, 360 mg over 4 h), with 100 mg hydrocortisone and 50 mg diphenhydramine premedication. The patient was clinically and vitally stable after receiving the dose, with no immediate reactions. Laboratory tests before administration of the first biologic dose revealed that his kidney function was normal. Two weeks after the initiation of IFX therapy, the patient presented with a 2 day history of generalized fatigue, nausea, and subjective fever with rigor. He had no history of vomiting, abdominal pain, diarrhoea, decreased urine output, hematuria, urine dribbling, difficulty passing urine, frothy urine, epistaxis, hemoptysis, shortness of breath, chest pain, or cough. He had no skin rashes, joint pain, nasal ulcers, recent contrast use, or recent infections, and had not used antibiotics, any other new drugs besides IFX, or herbal medications. In addition, the patient had no history of increased stoma output.

On physical examination, the patient’s vital signs were normal. He was fully conscious and showed no abnormalities in abdominal, respiratory, skin, joint, ocular, or neurological examinations. His laboratory test results showed the following values: serum creatinine, 671 µmol/L; urea, 36.2 mmol/L; HCO3, 8 mmol/L; and potassium, 5.7 mmol/L. Urine analysis revealed hyaline casts and no red blood cells, white blood cells, or proteins. The microalbumin level was 9.79 mg/mmol. The lactate levels were within the normal range. No abnormalities were observed in the complete blood count, including the eosinophil count, white blood cell count, and hemoglobin levels. The erythrocyte sedimentation rate and C-reactive protein level were within normal limits. Fecal calprotectin levels were negative. Blood and urine cultures were negative. ANA, C3/C4, anti-dsDNA, cANCA, pANCA, anti-GBM antibody hepatitis panel, syphilis antibodies, serum cyoglobulin, and anti-streptolysin tests were also negative. Beta2 microglobulin and N-acetyl beta-D-glucosaminidase unavailable. Computed tomography of the kidneys, ureters, and bladder (CT KUB) was performed to rule out obstruction and revealed no urolithiasis or obstructive uropathy.

**Fig. 1:**
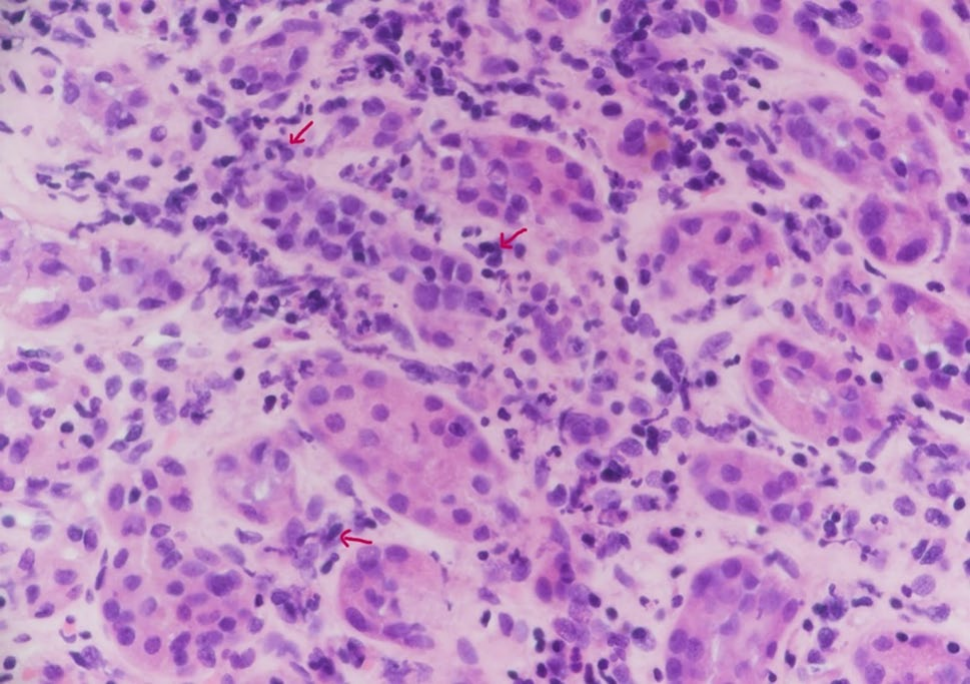
Light microscopic image of the renal biopsy specimen (hematoxylin–eosin [H&E] staining). Moderate interstitial inflammation composed of lymphocytes, plasma cells, and neutrophils (arrows). No crescents, fibrinoid necrosis, spike formations, and endocapillary hypercellularity can be observed

**Fig. 2:**
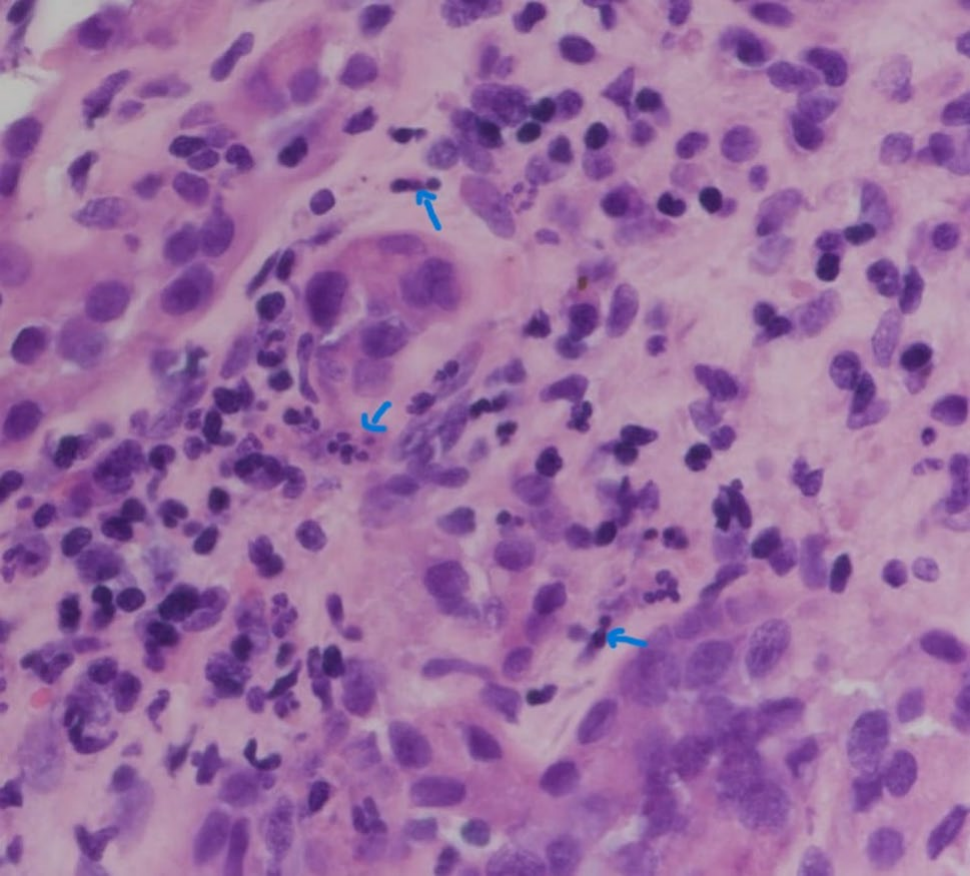
Light microscopic image of the renal biopsy specimen (H&E staining) Moderate interstitial inflammation composed of mixed inflammation and eosinophils (arrows)

After weighing the risks and benefits, the patient’s treatment was initiated with pulse methylprednisolone 15 mg/kg. Received 1 g of the drug on the first day of treatment. After the first dose of pulse therapy, 500 mg methylprednisolone was administered for 2 days. The patient’s serum creatinine level decreased from 672 µmol/L to 453 µmol/L; therefore, we continued with 1 mg/kg (60 mg PO OD) and a renal biopsy was performed (Figs. 1, 2). The biopsy specimen consisted of a core of renal tissue that contained the cortex and medulla. Twenty-seven glomeruli were present, none of which were globally sclerosed or exhibited segmental sclerosing lesions. Moderate interstitial inflammation composed of lymphocytes, plasma cells, neutrophils, and eosinophils, associated with mild tubulitis and acute tubular injury, was observed. Mild tubular atrophy and interstitial fibrosis (10–15% of the cortical tissue) were also noted. No crescents, fibrinoid necrosis, spike formation, or endocapillary hypercellularity was observed. The glomeruli showed a normal architecture, with patent capillary lumina and normal capillary wall thickness. No granulomas in the interstitium or the vasculature abnormalities were found. No deposition of IgG, IgG4 or C3 was observed in the tubular basement membrane. The pathological diagnosis was acute tubulointerstitial nephritis (ATIN). The patient continued 1 mg/kg (60 mg PO OD) for 2 weeks, tapper down steroids for 8 weeks, and the last serum creatinine level after 2 months of discontinuation of steroid treatment was 110 µmol/L. Renal impairment developed after the first dose of IFX and improved with steroid therapy, without any alterations in Crohn’s disease (CD) activity. This led to the conclusion that, in our patient, ATIN was not related to CD as an extraintestinal manifestation.

## Discussion

ATIN in IBD has been reported in patients receiving drugs, such as 5-ASA [3]. A systematic literature review identified nine cases of biopsy-confirmed AIN in patients with IBD treated with vedolizumab [4]. In addition to drug-induced causes, IBD is also a known cause of chronic granulomatous tubulointerstitial nephritis. Renal manifestations of IBD vary and include nephrolithiasis (calcium oxalate and uric acid), glomerulonephritis (IgA nephropathy and AAV), renal amyloidosis (AA), drug-related nephrotoxicity, and tubulointerstitial disease (acute tubular injury and tubulointerstitial nephritis) [2]. A few cases of IFX-induced ATIN have been previously reported [5, 14, 15] (Table 1).

**Table 1:** Infliximab Induce ATIN, case reports

Study: case report	country	time to onset of ATIN	Kidney biopsy	interventions	Needs Dialysis	outcome
Yoo et al. 2014 Infliximab-Induced Tubulointerstitial Nephritis with Image Findings of Striated Nephrogram in Crohn’s Disease	South Korea	2 years mesalazine had been maintained for 5.6 years without complication and renal biopsy showed active inflammatory response	There was diffuse tubular damage and infiltration of interstitial inflammation into the tubular epithelial cells Infiltration of various inflammatory cells, including lymphocytes, plasma cells, eosinophils, and a few neutrophils, was observed, and a higher proportion of eosinophil infiltration	Oral prednisolone Adalimumab, a fully humanized TNF-α antibody, was administered subcutaneously with mesalazine instead of infliximab, possibly to minimize immunologic reaction	No	The serum creatinine level showed partial recovery to 1.6 mg/dL after two weeks of steroid therapy, and stabilized from 2.6 mg/dl upon presentation with normal baseline (0.6 mg/dL)
Ota et al. 2016 Acute Tubulointerstitial Nephritis Associated with Infliximab in a Patient with Crohn’s Disease	Japan	9 years From 2003, the symptoms were successfully suppressed with the administration of IFX 6 mg/kg every 6 weeks in March 2012, the disease flared up again and was controlled with an increased dose of IFX (10 mg/kg))	Mononuclear cells, especially CD3 positive cells, and neutrophils infiltrated patchily in the interstitium and between the tubular epithelial cells. No granulomas in the interstitium or the vasculature abnormalities were found	Discontinue Infliximab During that period, was continued on mercaptoprine (20 mg/day)	No	One month later, creatinine clearance improved from 65.8 to 85.16 mL/ minute. Two months later, the CRP level and sCr went down to 1.48 mg/dL and 0.96 mg/dL respectively from 1.02 mg/dL and base line ( 0.85 mg/dl)
Tomonori Sato et. al.2018 Infliximab-Induced Tubulointerstitial Nephritis with Image Findings of Striated Nephrogram in Crohn’s Disease	Japan	4.5 years During maintenance therapy with agents including azathioprine and mesalazine, the intestinal symptoms of Crohn’s disease were stable	severe infiltration of inflammatory cells, including neutrophils, lymphocytes, plasma cells, and eosinophils, mainly in the interstitium and renal tubules	Discontinue Infliximab During that period, no relapse was noted while mesalazine administration was continued	No	Serum creatinine 1 mg/dl from 1.05 mg/dl and clinical symptoms were ameliorated after 2.5 months Contrast-enhanced CT showed several mild atrophic renal parenchyma lesions 9 months after the diagnosis
Alghamdi et al. 2024 Acute Tubulointerstitial Nephritis Associated with Infliximab Therapy in a Patient with Crohn’s Disease	Saudi Arabia	2 weeks from first dose of infliximab (5 mg/kg)	Moderate interstitial inflammation composed of lymphocytes, plasma cells, neutrophils, and eosinophils, associated with mild tubulitis and acute tubular injury, was observed	Pulse steroid, 1 g for 1 day then 500 mg for 2 days then 1 mg/kg 2 weeks, tapper down steroids for 8 weeks	No	serum creatinine level after 2 months of discontinuation of steroid treatment was 110 µmol/L from 671 µmol/L upon presentation

In our case, 4 months before the acute insult, the patient had a history of stage 3 acute kidney injury secondary to sepsis induced by a peripherally inserted central venous catheter line with full recovery of renal function upon discharge. The patient was then administered nephrotoxic agents, such as ciprofloxacin, for 14 days, 2 months before the acute insult and underwent contrast-enhanced CT. Laboratory test results before the biological treatment showed normal renal function.

Tumor necrosis factor-alpha inhibitors, such as IFX and adalimumab, are proinflammatory cytokines that are effective for moderate and severe CD and ulcerative colitis. Tumor necrosis factor is released by macrophages in the body during stress, and the binding of TNF to its receptors activates the immune system to secrete inflammatory cytokines and mediators. IFX inhibits TNF formation, leading to decreased inflammation and disease control. Reported renal complications of TNF-α inhibitors include drug-induced systemic lupus erythematosus [6, 7], AAV [8], and IgA vasculitis [8, 9].

As renal dysfunction in our case developed after the first dose of IFX and improved with steroid treatment without any altered CD activity, we concluded that ATIN was not related to CD. In the renal biopsy of our patient, mild interstitial fibrosis with monocytes, eosinophils, neutrophils and absence of granulomas supported the presence of acute interstitial inflammation due to the new agent, IFX. Ciprofloxacin was also not considered responsible for AIN because the patient’s renal profile was normal months after exposure to the drug and immediately before administering the IFX dose. The patient was not receiving any home medications for IBD; azathioprine was started for approximately 1 month after diagnosis and then stopped.

In a mouse model of lupus nephritis, interferon alpha-treated mice developed severe proteinuria and died of chronic glomerulonephritis (GN) and end-stage renal disease [10]. In previous case reports, ATIN developed after several administrations of IFX. However, in this case, ATIN developed after only one administration of IFX. Multiple doses received before developed manifestations of ATIN In all case reports of infliximab induced ATIN, In Ota et al. 2016 [5], developed after 9 years. Yoo et al. 2014 [14] reported after 2 years of administration of infliximab. Tomonori Sato et al. 2018 [15] reported that the timing of TIN onset after infliximab administration was 4.5 years. In our case report it is developed after 2 weeks from a first dose. The reason for late presentation in previous case reports remaining unknown. also, not mentioned in all three case reports regarding a form of infliximab. The fact that our patient received biosimilar IFX raises the question of whether the side effects were related to biosimilar IFX or to both types of IFX. A few reported cases of biosimilar IFX-induced liver injury have been diagnosed using liver biopsy [11, 12]. Regarding renal side effects, only one case of rheumatoid arthritis in a patient who received the biosimilar IFX and developed lupus nephritis has been reported [13]. it is needing further investigation if there is any relationship. Also, in pathology findings, it is important to differentiate by histopathology between Extraintestinal manifestations and drugs induced by presence of eosinophils supported drug induced.

## Conclusions

Although it is rare, infliximab could be a rare cause of elevated kidney function in IBD patents and that it should be not considered a completely safe treatment or disregarded as potential cause of ATIN specially in the absence of extraintestinal manifestations. Steroid can be effective management for infliximab-induced ATIN.
